# Development and Evaluation of a Serum Transfusion Process in the Thorny Devil Stick Insect (*Eurycantha calcarata*)

**DOI:** 10.3389/fvets.2022.847043

**Published:** 2022-04-04

**Authors:** Taylor M. Gregory, Ashlyn C. Heniff, Melinda A. Gorges, Andrew W. Lathan, Gregory A. Lewbart, Julie A. Balko

**Affiliations:** ^1^Department of Clinical Sciences, North Carolina State University, Raleigh, NC, United States; ^2^Department of Molecular Biomedical Sciences, North Carolina State University, Raleigh, NC, United States

**Keywords:** *Eurycantha calcarata*, serum, stick insect, transfusion, transfusion reaction

## Abstract

The thorny devil stick insect (*Eurycantha calcarata*) is a common invertebrate maintained under human care. Blood (hemolymph) transfusions are a widely used therapeutic tool in other species, but investigation in terrestrial arthropods remains scarce. Study objectives were development and evaluation of a serum transfusion process in the thorny devil stick insect. Twenty-five clinically healthy adult insects (9.9–23.0 g) were enrolled and baseline body weights were collected. Hemolymph collection was not successful in males, thus, all were recipient only (*n* = 12, MR). Females were divided into donor (*n* = 4, FD) or donor and recipient (*n* = 9, FDR) groups. Females were manually restrained and up to 1 mL of hemolymph was collected from the abdominal segment caudal to the proximal hindlimb using an 18 g hypodermic needle and passive collection *via* gravity. Hemolymph was quantified, centrifuged, and the serum separated. Insects were then injected superficially at the lateral aspect of the abdomen with 0.01 mL/g FD serum (MR), lactated Ringer's solution (LRS) equal to collected hemolymph volume (FD), or a combination of FDR serum and LRS equal to collected hemolymph volume (FDR). Response to stimulation, surface temperature, and righting reflex and mortality were serially assessed for up to 24 h and 7 days, respectively, following injection. In FD, median (range) injected LRS dose was 0.04 (0.03–0.06) mL/g. In FDR, median (range) injected serum, LRS, and combined serum and LRS dose was 0.03 (0.02–0.04), 0.01 (0–0.04), and 0.04 (0.02–0.06) mL/g, respectively. A mild temperature increase (maximum +2.9°C) (MR *n* = 10, FD *n* = 3, FDR *n* = 8) and delayed righting reflex (MR *n* = 4, FD *n* = 3, FDR *n* = 7) occurred in a subset of insects following injection. Two deaths occurred at 2 min (*n* = 1, FDR) and 96 h (*n* = 1, FD) post-injection. This is the first report of serum transfusions in thorny devil stick insects, and while largely successful, minor to severe transfusion reactions may occur.

## Introduction

The thorny devil stick insect (*Eurycantha calcarata*) is a member of the order Phasmatodea and is commonly cared for in both zoological institutions and research facilities. They are a tree and bush dwelling species and while native to Papua New Guinea, they are found in institutions throughout the world (1). This species is sexually dimorphic with females having a larger abdomen and males having larger spikes on the hindlimbs. Similar to many insects, *Eurycantha calcarata* are vulnerable to a range of health concerns including environmental diseases, infectious diseases, and neoplasia (2–5). Furthermore, as captive populations of invertebrates, including the thorny devil stick insect, increase, interest in and advancement of their medical care has grown (4).

A transfusion refers to administration of various blood (hemolymph) products to a subject for hemodynamic or cardiovascular support for the treatment of blood loss, blood component destruction, or lack of blood component production (6). Historically, whole blood transfusions were the sole product of transfusion medicine; however, advancements in technology and knowledge have expanded this repertoire to include specialized products such as packed red blood cells, plasma, cryoprecipitate, and platelet-rich plasma (6). Transfusion medicine has been extensively studied in companion animal medicine and research within the last decade has explored this topic in a variety of zoological species including reptiles, aquatic vertebrates, and terrestrial mammals (7–11). Despite the expansion of this realm into non-domestic species medicine, evidence-based data surrounding transfusion medicine in invertebrates and, specifically terrestrial arthropods, remains limited.

Similar to their use in vertebrate medicine, transfusions have the potential to support invertebrates through disease and/or complications from elective or emergent procedures, including hemolymph loss or dehydration. To the authors' knowledge, only two prospective transfusion studies have been performed in invertebrates and both have used Lepidoptera species. In moths, hemolymph of *Hyalophora cecropia* adults was injected into *Actias luna* larvae to investigate nutritional mechanisms of tissue morphogenesis (12). In the painted lady butterfly (*Vanessa cardui*), hemolymph was transfused between individual pupae to assess color pattern determination in butterfly wings (13). While the hemolymph collection and transfusion processes in both of these studies were successful, the methodologies are arguably not applicable to clinical invertebrate medicine. Additionally, given the tremendous biodiversity within the invertebrate taxa, extrapolation of transfusion protocols (e.g., technique, recommended volume, potential side effects) from one species to another should be practiced with caution. While anecdotal reports of invertebrate transfusions exist, to the authors' knowledge, no prospective studies have been performed in terrestrial arthropods and further exploration is warranted (14). The objective of this study was development and evaluation of a serum transfusion process in the thorny devil stick insect.

## Materials and Methods

Twenty-five clinically healthy, captive-bred adult thorny devil stick insects (12 male, 13 female) were obtained from a zoological collection and acclimated for 7 days prior to testing. Insects were group-housed by sex in a 36 × 24 × 36 cm ventilated enclosure with substrate (Eco Earth, Zoo Med Laboratories, San Luis Obispo, CA, USA), tree branches, and wild-harvested blackberry brambles, the latter replaced as needed to ensure ad lib access. Enclosures were kept at room temperature (20–22.2°C) and heavily misted with de-chlorinated water once a day to support a humid environment. Prior to study enrollment, all insects had a baseline physical examination performed by a veterinarian and were deemed clinically healthy. All insects were individually marked using non-toxic paint to allow individual identification throughout the study. Pilot testing prior to the study determined that hemolymph collection was unsuccessful in male thorny devil stick insects, thus, males were used as transfusion recipients only. Invertebrates are not covered under the North Carolina State University Institutional Animal Care and Use Committee (IACUC) and, as such, ethical approval was not obtained. However, standards comparable to those for the care and use of vertebrates were applied and all procedures were performed under the oversight of two veterinarians (GAL, JAB).

**Figure 1:**
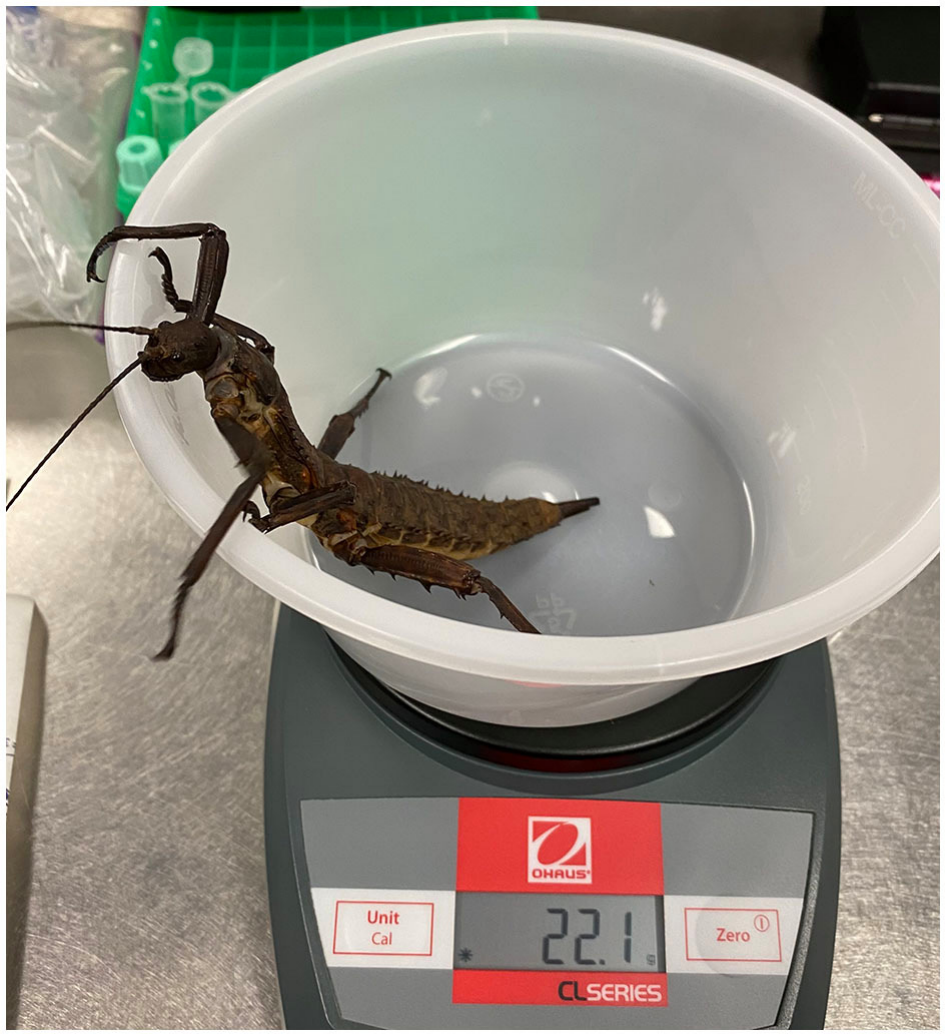
Body weight collection in a female thorny devil stick insect (*Eurycantha calcarata*).

On test day, all insects were manually removed from the group enclosure for visual examination and assessment of surface temperature (Milwaukee 10:1 Infrared Temp-Gun, Milwaukee Tool, Brookfield, WI, USA), response to stimulation, color, and righting reflex; a scoring rubric for the latter three characteristics was used and is described in Table 1. Righting reflex was assessed by manually placing the insect in dorsal recumbency and was considered normal, delayed, or absent if the insect successfully righted itself in <5 s, successfully righted itself in 5–30 s, or did not successfully right itself, respectively. Response to stimulation was assessed based on response to tactile stimulation associated with righting reflex assessment. Body weight (CL-201 Digital Gram Scale, Ohaus, Parsippany, NJ, USA) was also collected (Figure 1).

**Table 1:** Scoring rubric for assessment of response to stimulation, color change, and righting reflex prior to and following injection with homologous serum and/or lactated Ringer's solution in male and female thorny devil stick insects (*Eurycantha calcarata*).

**Variable**	**Score**	**Description**
Response to stimulation	*Responsive*	Normal movement or response to manipulation
	*Mildly unresponsive*	Mildly reduced movement or response to manipulation
	*Moderately unresponsive*	Markedly reduced movement or response to manipulation
	*Non-responsive*	No movement, unresponsive to manipulation
Color change	*Grade 1*	<25% discoloration
	*Grade 2*	25–50% discoloration
	*Grade 3*	50–75% discoloration
	*Grade 4*	75–100% discoloration
Righting reflex	*Normal*	Successfully rights itself in <5 s
	*Delayed*	Successfully rights itself in 5–30 s
	*Absent*	Unable to right itself in <30 s

During pilot testing, a quantifiable amount of anticoagulated hemolymph was unable to be collected from male or female stick insects. A technique for collection of coagulated hemolymph was developed, but was only successful in females. Thus, male stick insects were used as transfusion recipient-only and females were used as either donor-only or donor-recipient. For hemolymph collection, females were individually manually restrained with the right hindlimb held horizontal against the body segments. An 18 g, 1 inch hypodermic needle (Monoject, Covidien, Mansfield, MA, USA) was used to pierce the abdominal segment caudal to the proximal right hindlimb and hemolymph was allowed to drip *via* gravity into a graduated microcentrifuge tube (GeneMate 1.7 mL Graduated Microcentrifuge Tubes, Avantor, Radnor, PA, USA). Hemolymph was passively collected until 1.0 mL was obtained or a maximum of 90 s had elapsed and gentle pressure was then applied to the site using a 2 × 2 inch gauze square (Curity Sponge Gauze, Covidien, Mansfield, MA, USA). If at 90 s <0.5 mL of hemolymph had been collected, the contralateral (left) side was sampled using the described technique and the same microcentrifuge tube. The collected hemolymph was allowed to clot within the microcentrifuge tube and the total volume collected from each side was recorded. Following visible clot formation, the microcentrifuge tube was centrifuged (PowerSpin BX Centrifuge, United Products and Instruments Inc., Dayton, NJ, USA) for 10 min at 13,000 rpm, and the serum was aspirated and transferred to an individual, naive microcentrifuge tube using a 1 mL syringe and 25 g × 1 inch needle (Monoject, Covidien, Mansfield, MA). The volume of serum recovered from each female was recorded.

As previously noted, males were transfusion recipients only (MR, *n* = 12) and were designated to receive 0.01 mL of serum per gram of body weight (mL/g) using serum from an individual female donor (FD). Male body weights and collected female serum volumes were compared to identify a transfusion strategy that would minimize serum waste. This resulted in the designation of four females to serve as serum donors to all males (FD, *n* = 4). FD insects, in turn, received an injection of lactated Ringer's solution (LRS, B. Braun Medical Inc., Bethlehem, PA, USA) equal to the volume of collected hemolymph for that individual. The remaining females (*n* = 9) were designated to be serum donors and recipients (FDR) and, similar to above, body weights and collected serum volumes were compared to identify a transfusion strategy that minimized serum waste. While the sexual dimorphism of this species permitted use of a single female for serum donation to multiple males, females were only able to donate to a single female. Thus, FDR insects were designated to receive an injection of serum from a single identified female donor equal to the volume of hemolymph that had previously been collected from that recipient. If the identified donor could not provide the total target volume, the injection was supplemented with LRS.

Following serum collection but prior to injection, all insects were moved to individual clear, ventilated 21 × 13 × 7 cm containers (34 oz Storage Container, LocknLock, Seoul, South Korea) and baseline assessment was again performed including response to stimulation, color, surface temperature, and righting reflex. Target injection solutions—serum (MR), LRS (FD), or serum with or without LRS (FDR)—were prepared in 1 mL syringes with 29 g x 1/2 inch needles (U-100 insulin syringe, Exelint International, Redondo Beach, CA 90278). For injection, insects were briefly manually restrained in sternal recumbency and the needle was inserted at a shallow (170–175°) angle at the left lateral aspect of the abdomen between the third and fourth proximal segments. The needle was aspirated to confirm negative aspiration and the injection was subsequently performed over 5–10 s. Insects were assessed for reaction to injection, characterized by movement of the abdominal segment or limbs during injection.

Insects were assessed during injection and for two continuous minutes thereafter for any adverse responses. At 2 min, response to stimulation, color, and righting reflex were assessed and insects were returned to their individual containers. Insects were then serially evaluated for the aforementioned characteristics, in addition to surface temperature, at 5, 10, 15, and 30 min and 1, 2, 4, 6, 12, and 24 h following injection. At 24 h, insects were returned to group housing and subsequently visually and manually reassessed at 48, 72, 96, 120, 144, and 168 h post-injection for adverse clinical signs including death.

## Results

Median (range) body weight for insects in MR, FD, and FDR was 15.1 (9.9–19.3), 18.7 (17.8–19.7), and 18.8 (15.2–23.0) g, respectively. Median (range) baseline surface temperature in MR, FD, and FDR was 22.8 (22.2–23.1), 22.8 (22.5–22.9), and 20.6 (20.2–22.9)°C, respectively. Median (range) collected hemolymph volume for insects in FD and FDR was 0.8 (0.6–1.1) and 0.8 (0.5–1.0) mL, respectively. As a percentage of body weight, this translates to a median (range) of 4.2 (2.8–6.1) and 4.3 (2.4–6.1)% for FD and FDR, respectively. In FD and FDR, 3/4 and 2/9 insects required hemolymph collection from two sites (i.e., right and left) and this was not influenced by body weight.

In MR, median (range) injected serum dose was 0.01 (0.01–0.01) mL/g. In FD, median (range) injected LRS dose was 0.04 (0.03–0.06) mL/g. In FDR, median (range) injected serum, LRS and combined serum and LRS dose was 0.03 (0.02–0.04), 0.01 (0–0.04), and 0.04 (0.02–0.06) mL/g, respectively. Response to injection was observed in 5/12, 3/4, and 4/9 insects in MR, FD, and FDR, respectively. No color change was observed in any insect at any time point following injection. At the 2-min time point, mildly decreased response to stimulation was noted in 2/12, 1/4, and 1/9 insects in MR, FD, and FDR, respectively, and non-response to stimulation was noted in 1/4 and 1/9 insects in FD and FDR, respectively. At all subsequent time points, non-response to stimulation continued in the aforementioned insect in FDR with all other insects returning to baseline with a normal response to stimulation. Changes in righting reflex (normal, delayed, absent) following injection for insects in all groups are reported in Table 2. In general, 4/12, 3/4, and 7/9 insects in MR, FD, and FDR, respectively, had a delayed righting reflex at at least one time point following injection. Surface temperature changes (increase, decrease, no change) following injection for insects in all groups are reported in Table 2. In general, surface temperature was increased in 10/12, 3/4, and 8/9 insects in MR, FD, and FDR, respectively, at at least one time point following injection with the largest temperature change being +2.9°C in an FDR insect at the 2-h time point. From the 2-min through 2-h time point, the average group temperature remained increased compared to baseline in MR, FD, and FDR with a range of +0.01–0.6°C. From the 4-h through 24-h time point, the average temperature remained decreased compared to baseline in MR, FD, and FDR with a range of −0.04 to −0.6°C. At the 24-h time point, median (range) temperature change from baseline for insects in MR, FD, and FDR was −0.2 (−1.0 to 1.6), 0.3 (−0.1 to 1.2), and 0.6 (−0.2 to 1.0)°C, respectively.

**Table 2:** Number (percentage) of male serum recipient (MR), female serum donor (FD), and female serum donor and recipient (FDR) thorny devil stick insects (*Eurycantha calcarata*) with a change in surface temperature (increase, decrease, no change) and righting reflex (present, delayed, absent) prior to and following injection with homologous serum (MR, FDR) and/or lactated Ringer's solution (FD, FDR); ^*^denotes a missing value.

		**Temperature**			**Righting reflex**		
	**Group**	**Increase**	**Decrease**	**No change**	**Present**	**Delayed**	**Absent**
Baseline	MR			12 (100%)	12 (100%)		
	FD			4 (100%)	4 (100%)		
	FDR			9 (100%)	7 (78%)	2 (22%)	
2 min	MR				11 (92%)	1 (8%)	
	FD				3 (75%)	1 (25%)	
	FDR				6 (67%)	2 (22%)	1 (11%)
5 min	MR	7 (58%)	2 (17%)	3 (25%)	10 (83%)	2 (17%)	
	FD	1 (25%)	3 (75%)		2 (50%)	2 (50%)	
	FDR	8 (89%)	1 (11%)		4 (44%)	4 (44%)	1 (11%)
10 min	MR	7 (58%)	5 (42%)		12 (100%)		
	FD	2 (50%)	1 (25%)	1 (25%)	3 (75%)	1 (25%)	
	FDR*	7 (78%)	1 (11%)		4 (44%)	4 (44%)	1 (11%)
15 min	MR	8 (67%)	4 (33%)		12 (100%)		
	FD	2 (50%)	2 (50%)		3 (75%)	1 (25%)	
	FDR	8 (89%)	1 (11%)		4 (44%)	4 (44%)	1 (11%)
30 min	MR	4 (33%)	6 (50%)	2 (17%)	11 (92%)	1 (8%)	
	FD	3 (75%)	1 (25%)		1 (25%)	3 (75%)	
	FDR	8 (89%)	1 (11%)		4 (44%)	4 (44%)	1 (11%)
1 h	MR	2 (17%)	9 (75%)	1 (8%)	11 (92%)	1 (8%)	
	FD	1 (25%)	3 (75%)		2 (50%)	2 (50%)	
	FDR	7 (78%)	1 (11%)	1 (11%)	5 (56%)	3 (33%)	1 (11%)
2 h	MR	6 (50%)	4 (33%)	2 (17%)	11 (92%)	1 (8%)	
	FD		4 (100%)		4 (100%)		
	FDR	7 (78%)	2 (22%)		5 (56%)	2 (22%)	2 (22%)
4 h	MR		12 (100%)		12 (100%)		
	FD		4 (100%)		3 (75%)	1 (25%)	
	FDR	7 (78%)	2 (22%)		4 (44%)	4 (44%)	1 (11%)
6 h	MR	2 (17%)	9 (75%)	1 (8%)	12 (100%)		
	FD		4 (100%)		4 (100%)		
	FDR	7 (78%)	2 (22%)		6 (67%)	2 (22%)	1 (11%)
12 h	MR	1 (8%)	11 (92%)		12 (100%)		
	FD		4 (100%)		3 (75%)	1 (25%)	
	FDR	7 (78%)	2 (22%)		6 (67%)	2 (22%)	1 (11%)
24 h	MR	1 (8%)	11 (92%)		12 (100%)		
	FD		4 (100%)		3 (75%)	1 (25%)	
	FDR	7 (78%)	2 (22%)		8 (89%)		1 (11%)

Two deaths occurred during the study and included one FDR insect and one FD insect. Upon placement in dorsal recumbency for the 2-min righting reflex assessment, the FDR insect displayed several seconds of spastic limb movement followed by abrupt cessation of movement and no subsequent response to stimulation; this insect remained unresponsive at all subsequent assessments. The FD insect was discovered unresponsive at the 96-h assessment point. Gross necropsy of both insects revealed eggs in the coelomic cavity and no other significant findings.

## Discussion

This is the first prospective study and report of the use of serum transfusions in male and female thorny devil stick insects. The serum transfusion protocol developed and evaluated in the current study was subjectively easy to implement, repeatable between subjects, resulted in mild clinical side effects for both donors and recipients, and is readily applicable to a clinical setting.

Hemolymph collection was subjectively easy to perform in all females using an 18 g hypodermic needle and passive collection device. Only brief manual restraint was required for sampling and the chosen collection site was successful in all females, with only a small subset of subjects requiring collection from two sites. As noted, the technique was not successful when applied to males. While the ultimate reason for this is unknown, the markedly faster coagulation cascade of invertebrates compared to vertebrates and the smaller size of male thorny devil stick insects compared to females may play a role (15). The target collection volume (1.0 mL) was not reached in most females with a median collection volume of 0.8 mL. In general, non-terminal blood collection in veterinary species should be limited to <10% of circulating blood volume which, on average, is ~60–90 mL/kg in cats and dogs, respectively (16). Hemolymph volume varies considerably amongst insects and while this value for *Eurycantha calcarata* is unknown, hemolymph volume has been estimated to be 15% of body weight in the common stick insect (*Carausius morosus*) (17). Hemolymph collection volume in the current study ranged from 2.4 to 6.1% of body weight and this was largely tolerated in all females. That said, both females that died had the largest hemolymph volume collected (6.1%) and this may have contributed to their outcome. Until optimal hemolymph collection volumes are identified, lower collection volumes (i.e., < 5% of body weight) in the thorny devil stick insect should be targeted. Hemolymph collection was unsuccessful in males, a limitation of the current study. Sexual dimorphism and the smaller body size of males may have played a role although sex differences in hemolymph volume, the coagulation cascade, or the hematologic response to trauma cannot be ruled out. Hemolymph collection from male thorny devil stick insects may be possible with alternative sampling techniques and requires further investigation.

Following hemolymph collection, all donor-only female insects were injected with an equivalent volume of LRS to support effective circulating hemolymph volume. As noted, a single female in this group was found deceased at the 96-h time point. This female was the only insect of those still alive with a delayed righting reflex at the 24-h time point, potentially a reflection of subtle or early compromise. The delayed time course from hemolymph collection and LRS injection to death makes it less likely that these events were directly related, however, this cannot be ruled out. The consequence or outcome of hemolymph collection without subsequent fluid replacement in *Eurycantha calcarata* is unknown.

Homologous serum transfusions in male and female thorny devil stick insects were successfully performed in the current study. The target serum transfusion dose for male insects was 0.01 mL/g. This dose was extrapolated from companion animal medicine in which the plasma transfusion dose recommendation is similar at 10 mL/kg (0.01 mL/g) (18, 19). While this dose was well-tolerated in male insects in the current study with no deaths and mild side effects, the optimal serum transfusion dose in this species is unknown. Recall that, compared to female insects, males in the current study did not have hemolymph removed prior to injection, thus, dose recommendations for male insects with known hemolymph loss may be greater. In female insects, a target transfusion dose equal to the collected hemolymph volume was used and ranged from 0.02 to 0.06 mL/g. As females were both recipients and donors and, thus, had known hemolymph loss, higher target transfusion volumes were chosen compared to males. The administered serum dose range in females in the current study was 0.02–0.04 mL/g (20–40 mL/kg), 2–4 times the dosage used in male insects. While not definitively proven, the higher serum dose may have contributed to the greater incidence of side effects observed in female compared to male serum recipients in the current study. Logistical considerations (i.e., a single donor for each recipient) and collection volume limitations necessitated the addition of LRS to all but one female serum transfusion; this created an added confounding factor in the current study.

While transfusion medicine has advanced significantly in the past several decades, there are still risks and potential complications associated with transfusion administration. Reactions to transfusions can range from mild to severe and can, in some circumstances, result in death. Clinical signs of transfusion reactions in companion animals may include changes in respiratory character (tachypnea, dyspnea), cardiovascular disturbances (hypotension, tachycardia), pyrexia, facial edema, chemosis, and/or anaphylaxis (20, 21). In invertebrates, clinical signs of transfusion reactions have not been described. The current study attempted to use changes in response to stimulation, righting reflex, surface temperature, and color as well as mortality to characterize transfusion tolerance. Response to stimulation was only briefly altered in a small subset of insects from all groups and, in light of this timing and scope, may represent a response to handling and/or the injection itself. A delay in righting reflex was also observed, however, this finding, in comparison, affected the majority of insects at one or more time points throughout the 24-h assessment period. The magnitude of this side effect waned over time with only one insect in FD having a delayed righting reflex at the 24-h time point. Similar to above, identification of this side effect in donor-only insects suggests that it may not be specific to serum transfusions in thorny devil stick insects. The reason for the delayed righting reflex in two female insects in FDR prior to injection is unknown, but both had a normal righting reflex by the 24-h time point.

In the current study, an increase in surface temperature, up to 2.9°C, was observed in male and female insects following serum administration. Possible differentials for this temperature increase include a transfusion reaction, a response to stress/handling, or changes in environmental temperature. All testing was conducted in a climate-controlled room, thus, surface temperature increases secondary to increased environmental temperature, although not impossible, were less likely. Additionally, if increased temperatures were due to changes in the environment or handling stress, this effect would theoretically be consistent across all groups. Female donor-only insects demonstrated an inconsistent pattern of mild temperature fluctuations with a decrease in temperature in all subjects from the 2-h time point onward, making environmental or handling factors less likely. Furthermore, no clinically apparent consequences to this temperature change were appreciated and the majority of insects exhibited a decrease in temperature in the latter portion of the assessment period.

The most severe potential transfusion reaction in the current study was the unresponsiveness and death of one female serum transfusion recipient immediately following injection. Although a definitive cause of death is not known and gross necropsy was inconclusive, the time course would support a transfusion-associated death. As mentioned previously, this female had the highest percentage of hemolymph collected and this may have also been a contributing factor. Overall, this death results in a serum transfusion mortality rate of 4.8% (11.1% female, 0% male) for thorny devil stick insects in the current study. Aside from the single, acute mortality event, none of the assessed characteristics proved a definitive association with a transfusion reaction. Recalling that no other clinical side effects were appreciated throughout the assessment period, this supports the notion that serum transfusions are largely well-tolerated in this species, with the caveat that clinical assessment of this species is challenging and more subtle signs of a transfusion reaction may not have been clinically apparent. On a similar note, transfusion reactions have been reported in companion animals for up to 12 days following administration, thus, late transfusion reactions beyond the 7-day assessment period may have been missed (20, 21).

At this time, the impact of various factors including genetics, sex/hormones, age, environmental temperature, and the invertebrate-specific phenoloxidase cascade on the success of thorny devil stick insect transfusions remain unknown and warrants future investigation. Additionally, as the components and roles of invertebrate hemolymph are unique compared to vertebrate blood, the clinical use and application of transfusions in invertebrates may ultimately vary compared to their vertebrate counterparts (22).

In general, serum transfusions from female to both male and female thorny devil stick insects in the current study were well-tolerated and observed side effects did not result in clinically significant or apparent consequences. While insects were assessed for 7 days following transfusions, the long-term clinical consequences of serum transfusions in this species are unknown. It should also be noted that study subjects were clinically healthy insects and the use of serum transfusions in diseased or compromised individuals may result in different outcomes. As with any species, the risk-benefit ratio and corresponding clinical judgement should be carefully weighed when considering the use of a serum transfusion in a thorny devil stick insect or other terrestrial arthropod.

## Conclusion

In conclusion, hemolymph can be successfully and easily obtained from female *Eurycantha calcarata* adjacent to the proximal hindlimb using manual restraint, a hypodermic needle and passive collection. Robust collection volumes (>5% of body weight) may increase the risk of mortality and should be avoided. Serum transfusions were successfully performed in male and female thorny devil stick insects in the current study using median doses of 0.01 and 0.03 mL/g, respectively. Although two deaths occurred, the remaining side effects (increase in surface temperature, delayed righting reflex) were mild, clinically insignificant, and occurred in both donors and recipients. Serum transfusions can be considered for use in this species and monitoring for transfusion reactions is recommended.

## Data Availability Statement

The original contributions presented in the study are included in the article/supplementary material, and further inquiries can be directed to the corresponding authors.

## Author Contributions

TG, GL, and JB contributed to study conception. TG organized the data. All authors contributed to study design and data collection and contributed to data analysis, manuscript creation, and read and approved the submitted version.

## Conflict of Interest

The authors declare that the research was conducted in the absence of any commercial or financial relationships that could be construed as a potential conflict of interest.

## Publisher's Note

All claims expressed in this article are solely those of the authors and do not necessarily represent those of their affiliated organizations, or those of the publisher, the editors and the reviewers. Any product that may be evaluated in this article, or claim that may be made by its manufacturer, is not guaranteed or endorsed by the publisher.
